# Pathways to inclusive and equitable quality early childhood education for achieving SDG4 goal—a scoping review

**DOI:** 10.3389/fpsyg.2022.955833

**Published:** 2022-07-22

**Authors:** Dana Rad, Adela Redeş, Alina Roman, Sonia Ignat, Raul Lile, Edgar Demeter, Anca Egerău, Tiberiu Dughi, Evelina Balaş, Roxana Maier, Csaba Kiss, Henrietta Torkos, Gavril Rad

**Affiliations:** ^1^Faculty of Educational Sciences, Psychology and Social Work, Center of Research Development and Innovation in Psychology, Aurel Vlaicu University of Arad, Arad, Romania; ^2^Academy of Romanian Scientists, Bucharest, Romania; ^3^Universitatea Hyperion din Bucureşti, Bucharest, Romania

**Keywords:** scoping review, inclusive and equitable quality early childhood education, SDG4.2, micro level, meso level, macro level

## Abstract

According to Sustainable Development Goal 4.2 (SDG 4.2), Equal Access to Quality Pre-primary Education, governments throughout the world are working to ensure that all children have access to high-quality early childhood development, care, and pre-primary education by 2030. In order to organize available evidence into a coherent framework, the current scoping review represents an exploratory synthesis addressing the broad question of what qualitative and inclusive Early Childhood Education and Care strategies are currently being established globally to achieve SDG4 targets. The goal of this scoping review in this respect, was to map the available research and offer an overview of micro-, meso-, and macro-level perspectives on evidence-based interventions and strategies, for the promotion of SDG4 globally. A layered model of early childhood education that is both inclusive and egalitarian education emerged, starting with the micro level: child, family and community, mezo level: nursery, and kindergarten and macro level: national policies and SDG 4.2 Agenda for 2030. The mezzo level connects the micro and macro levels, being the most solicited level of implementing inclusive and qualitative ECEC strategies. Thus, starting with putting a real emphasis on children rights, creating a qualitative and inclusive culture with a holistic understanding of child development, then investing in teacher preparation and instilling a strong belief and positive attitudes toward equity in early childhood services, developing inclusive educational policies with an authentic community support offered by all stakeholders, then adapting curriculum and assessment methods to all early childhood educational contexts and lastly piloting and up-scaling good practices, and investing in infrastructure, facilities and innovative educational services, SDG4.2 targets could transparently and efficiently be attained by 2030, with all the setbacks arisen from the pandemic context. The data provide light on a vast topic range, including human rights and values, policy actions, and ideologies. The micro-level themes emphasized the importance of fostering equitable and inclusive environments for children., as well as instructional approaches that encourage positive attitudes toward diversity and instructors' levels of experience in dealing with diversity. We also discovered the significance of creating chances that promote socialization, connection development, and a sense of belonging. Meso-level principles emphasized the relevance of schooling in a child's holistic development and skill acquisition. Mainstream availability for all children, national curriculum regulations, teacher preparation for inclusive early childhood education, excellent funding and governance, evaluation and monitoring, and research on inclusive early childhood education comprise the macro level. As a concept and an approach, inclusive and qualitative education necessitates the preparedness of all relevant educational components to participate. Providing inclusive education in the early years requires setting the foundation for subsequent levels of schooling. The active engagement of a young kid should be directed by developmentally and individually suitable curricula. Access to and participation in age-appropriate general curricula becomes critical in identifying and providing specialized support services. Inclusive programming does not imply that the educational programs will necessarily be of good quality. Efficiency and wellbeing are synonymous with equity. Equitable education investment benefits everyone in society, not just the most marginalized. Investing in education will help communities achieve all of the Sustainable Development Goals related to education.

## Introduction

Human rights to education are both essential and beneficial. To actualize this right, governments must ensure that everyone has free and obligatory access to high-quality, inclusive, and equitable education and learning, with no one being left behind. Mutual understanding, tolerance, friendship, and peace, as well as the complete development of the human personality, should be the goals of education.

When the Millennium Development Goals (MDGs) were completed in 2015, it was obvious that tremendous progress had been made toward universal primary school completion throughout the world, but that a trend in development among the most disadvantaged had come to a halt (Ortigara et al., 2018). Simultaneously, it became evident that access was insufficient for learning, with an estimated 250 million youngsters lacking critical abilities, regardless of whether they were in school or had completed up to 4 years of elementary school (Ortigara et al., 2018). As a result, promoting educational quality, inclusion, and fairness in and through education are crucial components of the SDG4 aim, and monitoring progress until 2030 is essential (Friedman et al., 2020). As a result, reducing educational disparity is one strategy to promote a more equitable distribution of human capital and the formation of more equitable human societies.

Early childhood development, care, and education involve a wide variety of aspects affecting a child's wellbeing, such as physical, social, emotional, and mental health (Blackmore et al., 2016; Ackah-Jnr and Fluckiger, 2021). In general, growth continues *via* a succession of predictable and normal stages: as children get older, they become more self-sufficient and develop more complicated abilities and capabilities (Boeren, 2019). Children, on the other hand, develop at varying rates and may reach developmental milestones at different times. Because parental expectations and practices range not just among nations, but also across cultural, ethnic, and religious groups within the same country, what is considered normal child development varies by culture and environment (Ward, 2014; Sira et al., 2018; Piggott et al., 2021). All children have an inherent right to perform at a high level, regardless of their growth rate or pace.

In the majority of countries where data is available, more than half of children aged 3 to 4 are developmentally on track (Silva, 2017; Do et al., 2020; Kinkead-Clark et al., 2020; Lorente et al., 2020). In all countries with comparable statistics, more than 85% of children aged 3 to 4 are seen to be on pace with their physical development. In terms of academic and social-emotional development, the number of children who are on track varies greatly, although it is higher than 50% in almost every country (UNESCO Institute for Statistics, 2021). Children are the least likely to be considered developmentally on track in the domain of literacy-numeracy among all nations with accessible data (Class, 2020; Tatto et al., 2020).

A new study demonstrated the influence of trauma on cognitive development, such as that encountered in conflict situations, as well as the need of providing safe and stimulating learning environments (Nelson and Carver, 1998; Enlow et al., 2012). Investing in high-quality holistic early childhood development, care, and education for children of all ages is an important method for enhancing learning and decreasing inequality, especially for the most vulnerable children (Richard, 2019; Beeharry, 2021). It is proposed that at least 1 year of excellent pre-primary education be given free and required by well-trained instructors (Gilor and Katz, 2017; Prinz and Kulik, 2018). This should be done while keeping in mind the realities of various nations, including their capacities, levels of development, resources, and infrastructure. Investing in young children, particularly those from disadvantaged backgrounds, has the greatest long-term value in terms of developmental and educational outcomes (Spencer et al., 2005; Hanline and Correa-Torres, 2012; Meeks and Jain, 2015). Parents, health care providers, and educators may better plan, design, and implement timely interventions to meet the needs of children with disabilities, reducing developmental delays, improving learning outcomes and inclusion, and minimizing marginalization (Spencer et al., 2005; Hanline and Correa-Torres, 2012; Meeks and Jain, 2015; Unterhalter, 2019; Addey, 2021).

To guarantee that no one falls behind, all students, regardless of background or handicap status, require appropriate physical infrastructure and safe, inclusive learning settings (McDevitt, 2021). Children engagement in school is severely limited due to a lack of consideration to their rights and needs (Thamminaina et al., 2020).

Teachers are essential to achieving all of the SDG4 goals (Unterhalter, 2019). Countries will need to hire more teachers who are empowered, appropriately recruited and paid, motivated, properly equipped, well-trained, and supported in order to face the challenges of universal primary and secondary education (Unterhalter, 2019). The distribution of instructors within countries is very uneven. Fairness in education must be achieved in rural and urban areas, across sub-regions, and within and between institutions (Mahlo, 2011; Piggott et al., 2021).

When instructors in inclusive schools think they are competent of instructing all students, according to academics, inclusive education has the ability to change the way children learn (Florian, 2015; Florian et al., 2017). According to research, the most significant barrier to inclusive education is instructors' misunderstanding of the notion of inclusive teaching (Florian, 2015; Florian et al., 2017; Saloviita, 2018). Florian (2015) defines inclusive teaching as the pedagogical act of fostering equal and engaging learning environments in which learners may develop as entire people through flexible learning and evaluation regimes. Teachers in inclusive education value student diversity and think that with the correct support, any kid can learn (Allan, 2008).

With inclusive teaching, you may challenge the traditional perspective of fixed skills and see yourself as a transforming actor in the teaching and learning process (Florian, 2015). According to previous research, inclusion teaching typically ignores traditional pedagogy and it is only concerned with incorporating special educational activities into the mainstream education (Allan, 2006, 2008; Nilholm and Alm, 2010).

Inclusive pedagogy focused educators focus on learning as a common activity rather than individual distinctions among learners. When teachers, students, and other support workers study as a group, they collaborate to create an authentic learning community (Allen and Cowdery, 2012; Florian, 2015). Nothing pays off more than interacting publicly and respectfully with children, including those with impairments (Jones, 2014). Inclusive teaching is a cognitive activity that requires adequate pedagogical abilities and suitable student help, as well as the use of technology and the formation of respectful relationships that result in pleasant encounters (Klibthong and Agbenyega, 2013).

Children's educational potential are being eroded by social tensions, sickness, and catastrophes. In war and disaster situations, many of the most serious educational deficiencies may be identified (Deng, 2003; Kreso, 2008; Siriwardhana et al., 2013; Pascapurnama et al., 2018). In the face of war, social tensions, and natural disasters, it is vital to construct more resilient and responsive education institutions. Attainment, forced recruitment, kidnapping, and sexual assault must all be outlawed at educational institutions, as well as on the roadways that go to and from them. Better education is also necessary for avoiding and resolving disputes and crises, as well as fostering peace. Fulfilling SDG 4 in the context of protracted war, such as the Syrian and Ukrainian crises, as well as large-scale forced migration, has proved extremely difficult for governments and civil society organizations, according to studies on migrant and refugee early childhood education (Albakri and Shibli, 2019; Kalinina et al., 2022).

Averett et al. (2021) looked at how early childhood managers built facilities that were welcoming to gay and lesbian families, which is a sensitive topic in early childhood education. Only a few lesbian and gay affirming actions were found among the data, which demonstrated a spectrum of behaviors biased toward heterosexism and homophobia. More training and policies in early childhood settings that are tolerant of lesbian and gay led families are all consequences.

Battaglia and Lebedinski (2015) Roma population research provides further evidence-based studies with disadvantaged youngsters. This study looks at the Roma Teaching Assistant Program, a remedial education program aimed at Serbia's socially oppressed Roma community. Although the impact on dropouts or grades in all grades was not shown, a study of heterogeneous impacts found that students in the 1st grade benefitted in a larger proportion from the program than older children, with reduced dropout rates and higher grades. Overall, the data affirmed that focused remedial education programs can enhance the chances of low-achieving youngsters (Battaglia and Lebedinski, 2015).

While the gap in educational access persists, the difference in educational quality and learning results is becoming a growing concern for education (Friedman et al., 2020). Quality, equity, and inclusion are central to SDG 4 (McDougall, 2016; Unterhalter, 2019), however education quality varies widely and tends to reinforce marginalization and discriminatory processes, with the most privileged having access to much higher-quality education than the least privileged (Friedman et al., 2020). This is worsened by the rise of private and fee-paying schools, which essentially exclude people who cannot afford them (Abbate-Vaughn et al., 2011). At the same time, many of these private providers operate in an unregulated environment with limited quality assurance. Education is a government-provided service for which the government is accountable. To achieve SDG 4 and ensure that no one lags behind, governments must ensure that education is publicly funded and managed (Unterhalter, 2019; Friedman et al., 2020). Governments must guarantee that private players follow public norms and, if this is not practicable, do not manage educational institutions for profit.

To overcome gaps in evaluating equity and inclusion as well as quality and learning outcomes, more nationally and internationally measures are required (Caro, 2021). For a better assessment and quality monitoring, governments' capacity to disaggregate data properly and use it effectively for planning and policymaking should be strengthened. Governments' capacity to track wider learning outcomes must be improved (Milovantseva et al., 2018). Better statistics on especially vulnerable populations, as well as results are required (Beeharry, 2021). Through the development of knowledge, skills, values, and attitudes, the new global education agenda aims to build citizenship, resilience, empathy, tolerance, and sustainability through the development of knowledge, skills, values, and attitudes (Edwards et al., 2020; Pashby and Sund, 2020; Beeharry, 2021). The process is tough when it comes to measurement.

Capacity constraints must be addressed in order to ensure that excellent education is accessible and equitable. Performance is intrinsically tied to increased educational investment and enhanced government capabilities. To guarantee that no one falls behind, capacity training and investment in efficient use of public money, gender mainstreaming, disaggregated data collection and analysis, evidence-based policy making and planning, and monitoring equality and inclusion are all essential. Diverse stakeholders should collaborate to provide governments with coordinated technical help to meet their capacity needs (Galkiene and Monkeviciene, 2021).

## Rationale of the present scoping review

The widespread disruption of children's education as a result of educational institutions closures due to public health concerns has been one of the most devastating repercussions of the COVID-19 epidemic. According to the UNESCO Institute for Statistics, the pandemic will affect about 100 million children in eight age groups who do not meet the reading competency standard by 2020 (Bronfenbrenner, 2005; UNESCO, 2020; UNESCO Institute for Statistics, 2021). Closures of COVID-19 threaten exacerbated vulnerabilities for already vulnerable persons (Azevedo et al., 2021), a deeper lack of social involvement (Larsen et al., 2021), and a quickly expanding social inequality scenario (Fredman, 2021). Research (Orsander et al., 2020) was done among Save the Children program participants in 37 countries throughout the world. In this worldwide sample, there are 16,110 children, with around 15% under the age of 4 and 30.6% between the ages of 5 and 10. A disability was reported by 3.9% of those in this group. The COVID-19 epidemic, according to the findings of this study, has expanded the distance between disabled children and their parents.

The current scoping study is a preliminary assessment of the amount and scope of existing research literature on evidence-based interventions and methods for global SDG4.2 promotion. The current scoping review is an exploratory synthesis addressing the broad question of what qualitative and inclusive Early Childhood Education and Care strategies are currently being implemented globally to achieve SDG4 goals and offers an overview of microlevel, mesolevel, and macrolevel perspectives in order to organize available evidence into a coherent framework.

Although there is a wealth of literature on strategies, practices, and interventions for addressing the issue of providing high-quality, inclusive Early Childhood Education and Care (ECEC) services in kindergartens and nurseries, there is no systematic evidence of the impact of such isolated initiatives globally.

Beginning with Boeren (2019) study on understanding SDG 4 on excellent education, three integrated levels of action emerged: microlevel, mesolevel, and macrolevel perspectives, as shown in Table 1.

**Table 1:** SDG 4.2. targets: micro level, meso level, and macro level perspectives according to Boeren (2019).

**2. “Ensure that all girls and boys have access to high-quality early childhood development, care, and pre-primary education by 2030 so that they are prepared for primary school” (SDG 4.2)**		
**MICRO LEVEL**	**MESO LEVEL**	**MACRO LEVEL**
Parents who send their children to preschool and utilize the services provided; parents who understand the relevance of these activities.	Educating parents about the services that are available and presence of high-quality preschool and childcare programs in their neighborhood.	Preschool and childcare should get enough assistance from responsible governments, as well as efforts aimed at raising parental awareness about the benefits of preschool activities.
**PARENTS AND FAMILIES**	**KINDERGARTENS AND NURSERIES**	**GOVERNMENT AND POLITICS**

According to Boeren, individuals must comprehend the relevance of education and training in relation to the possible advantages they may give at the micro level (2019). In terms of SDG 4.2, it may be argued that parents all over the globe need to cultivate a positive attitude toward education and appreciate the benefits it can bring for their children, so boosting their own levels of agency, which are often limited by the systems in which they exist. These activities might be the result of attitudes, confidence, and drive developed earlier in life. When they become parents and have to send their own children to school, this may come in helpful. Naturally, none of this is feasible without a plentiful supply of qualitative education, which is a recurring issue at the mesolevel. In principle, food should be available within a reasonable distance of everyone's residence. In terms of pre-primary (SDG 4.2) education, this is projected to be substantial (ECEC). In order to reach the SDG goals, educational institutions must diversify their educational offerings. Countries with low adult learning participation rates, according to research (Boeren, 2019), have a limited supply of high-quality services. Educational leaders must work with their own personnel to promote inclusiveness, which is a theme that goes across all of the SDG 4 aims.

## Methodology

Like systematic reviews, scoping reviews establish eligibility criteria, perform literature searches, screen the results, and choose evidence for inclusion. The reliability of scoping reviews as a review technique for various indicators is growing. A scoping review will have a broader emphasis on a review issue than typical systematic reviews and proportionately more open-ended inclusion criteria. The difference between scoping reviews and systematic reviews is considerable. Researchers who perform systematic reviews first learn more about the amount of scientific literature on a certain issue (Centobelli et al., 2020; Ertz and Le Bouhart, 2022; Ertz et al., 2022); in our instance, this was done to learn more about prior research on both inclusive and qualitative ECEC approaches for achieving SDG4.2 goals.

We used this technique to classify results into categories and to identify gaps in the knowledge on qualitative and inclusive ECEC methods for achieving SDG4.2 targets (Sargeant and O'Connor, 2020). Our primary motivation for conducting this scoping review was to determine whether or not it would be beneficial to conduct a systematic review on the subject of qualitative-inclusive early childhood education. The quality and inclusiveness of the strategies used in ECEC are two issues that can be addressed simultaneously. Before doing a systematic review on the subject to further examine the efficacy of the treatments presented in the scientific literature, it is important to understand that high-quality education does not always equate to inclusive education and vice versa.

The purpose of the present scoping review is to locate and synthesize published research conclusions on inclusive and equitable evidence-based early childhood education programs. The following questions were the subject of the review:

1. What research methods have been utilized to look into evidence-based, inclusive, and equitable early childhood education initiatives?
2. What research instruments have been utilized to investigate excellent early childhood education efforts that are inclusive and equitable?
3. What outcomes have been recorded in terms of excellent early childhood education efforts that are inclusive and equitable?

Arksey and O'Malley (2005) technique and recommendations for conducting systematic scoping reviews was improved upon by Levac et al. (2010). To improve the technique, Levac et al. suggested expressing the research topic clearly and connecting the objective and research questions (phase one); integrating the feasibility of the scoping process with the breadth and scope of the scoping procedure (phase two); using a team-based iterative approach to study selection (phase three); extracting data (phase four); including a quantitative summary and qualitative thematic analysis in the report, as well as analyzing the implications of the findings for policy practice or research (phase five); finally, as a mandated implementation of evidence-based component of the scoping process, include dialogue with stakeholders (phase six) (Levac et al., 2010).

The search strategy aimed to locate previously published publications till April 15, 2022. To prepare for the search, the elements of a PICOC framework (population, intervention / exposure, comparison, outcome, and context) were used to identify the primary ideas contained in the research topics (Levac et al., 2010).

Three key principles were created for the creation of search methods. Population: preschool teachers, preschool parents, and educational decision-makers; interest: evidence-based Early Childhood Education and Care initiatives; and context: SDG4.2 were the themes.

A three-stage search strategy was employed, including Google Scholar and Dimensions AI plus with full text (EBSCO-host), as well as a review of text terms identified in titles and abstracts, and a study of the index keywords used to define each article. The second search was created and customized for the Web of Science database, including all of the observed keywords and index phrases. The searches were conducted using a building block search strategy (Moher et al., 2015). Each PICO main word was represented by a block of keywords/single words/control nouns. OR was used to merge individual search phrases inside the same block. Among the top search phrases were early childhood, inclusive education, excellent education, and SDG4. After the study was located, the reference lists of all included publications were combed for further material. Citations were found using Google Scholar, Scopus, Dimensions AI, PubMed, and Web of Science. The searches were conducted between April 3 and April 10, 2022.

**Figure 1:**
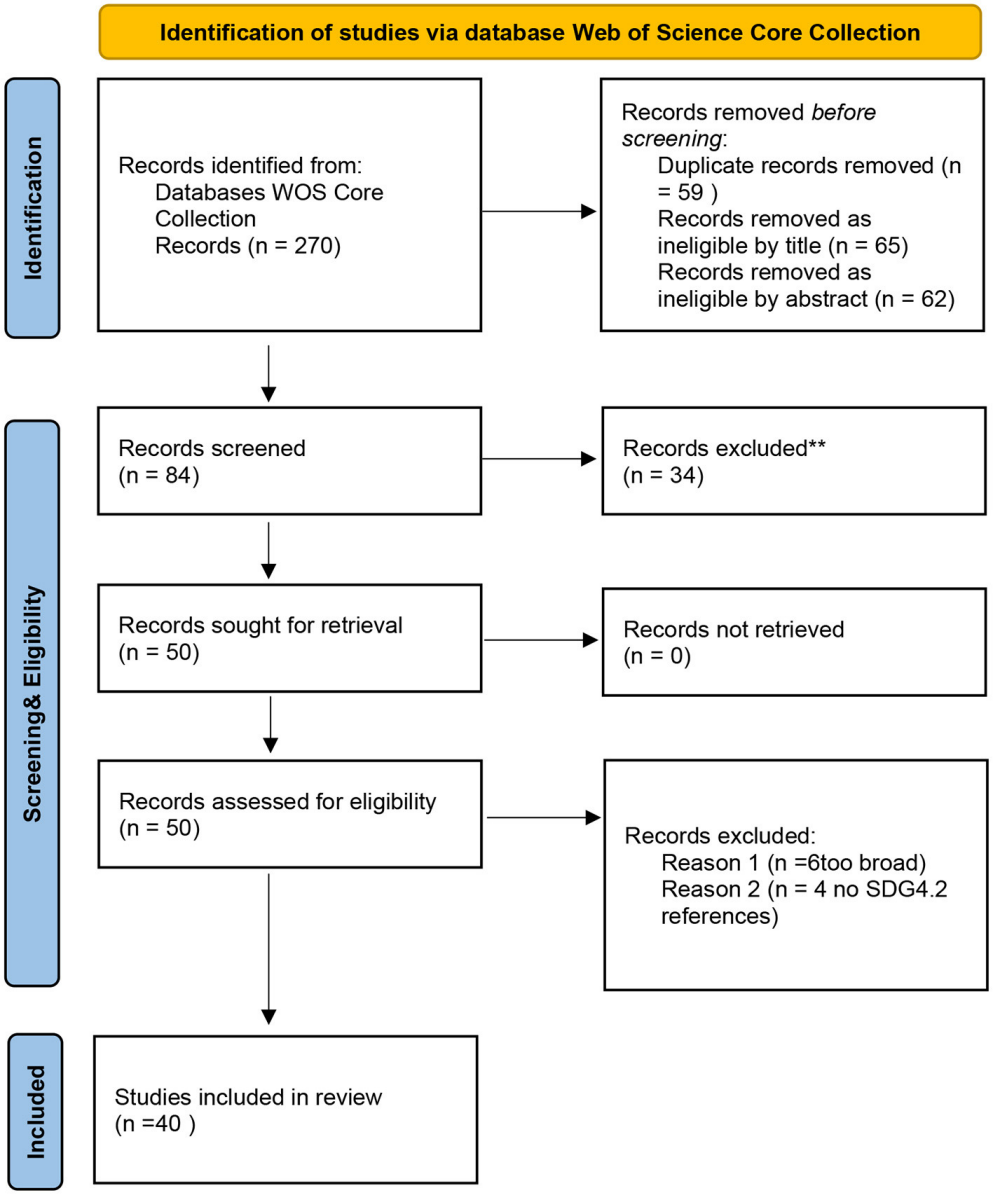
PRISMA flow diagram for inclusive and equitable quality early childhood education for achieving SDG4 Goal.

According to Moher et al., Figure 1 depicts a PRISMA flow diagram from search to final study inclusion (2015) (see Figure 1.

After searching the Web of Science Core Collection electronic database the investigations indicated by the studies contained in WOS, the found articles were converted into an excel file for title and abstract screening. Studies were only evaluated for inclusion if they fulfilled the review's inclusion and exclusion criteria. The review omitted studies that were not primary research, studies published in languages other than English, and publications that did not investigate on inclusive and equitable quality early childhood education. The team convened in accordance with the screening procedure standards to examine study inclusion and exclusion choices. Each manuscript was evaluated separately by two reviewers, with any differences handled during the screening process. To identify which studies to further process in order to answer the scoping review's questions, an excel data extraction sheet was created. The authors, publication year, country of origin, study design, study purpose, population, and research tools used to assess inclusive and equitable quality early childhood education were evaluated and compiled (Levac et al., 2010).

**Figure 2:**
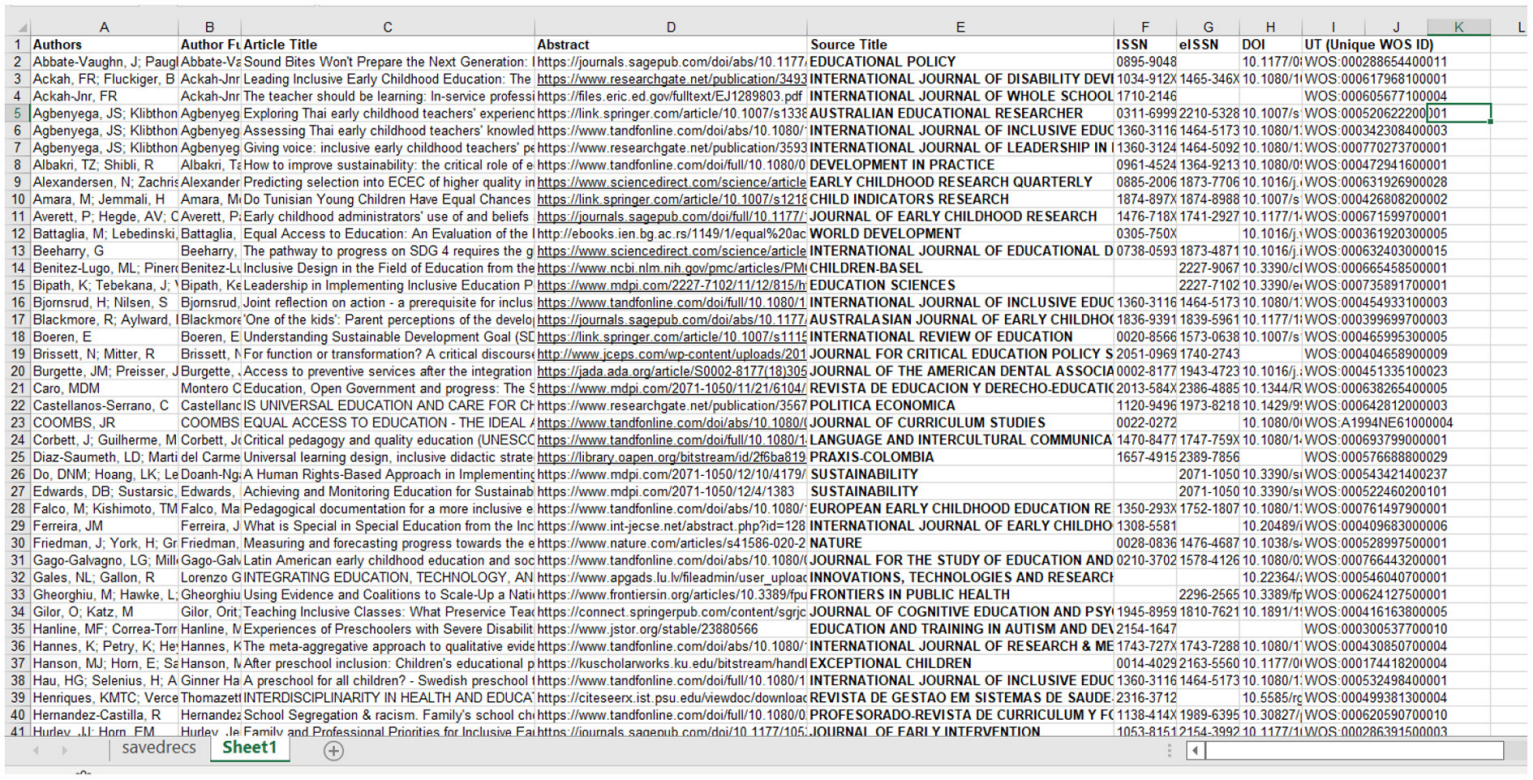
Sample of Studies on qualitative and inclusive ECEC strategies.

Figure 2 provides a sample of 41 studies included in our methodology highlighting their author(s), year of publication, objectives, methodology, and results, like presented in the systematic review methodology (Ertz et al., 2022).

Two reviewers extracted data and discussed it with four additional reviewers. Data abstraction-related uncertainties and conflicts were resolved through dialogue.

## Results

### Micro level

Positive social engagement, participation in everyday activities, a child-centered approach, tailored evaluation for learning and accommodations, adoptions, and support are examples of micro-level processes. They have an immediate influence on a child's sense of belonging, engagement, and learning.

It is critical to focus on families as the initial and primary setting for children's school and socio-emotional development. At the moment, there is a continuing battle to figure out how to effectively serve and assist an increasingly diverse community of families and children enrolled in ECEC programs. Family involvement in early education is defined as a technique used to form authentic relationships with families that promote overall family wellbeing and children's healthy development, as well as that builds partnerships with families for a common cause—-helping children grow and flourish. Family interactions is progressively being recognized as a key element of high-quality early care and education programs (Fantuzzo et al., 2013).

Jerome Bruner (1975) shown how poverty influences the raising of the very young and how different types of rearing impact human development. The developmental sciences have flourished since Bruner's groundbreaking publication. Neuroscience, genetics, and cognitive psychology discoveries have resulted in descriptions of brain, language and cognitive development, and more recently, resilience development. The majority of such findings are on child development, however they generally come from laboratory or family research. However, there is a growing corpus of study on the subject on how surroundings outside the home may help or impede a child's development.

A qualitative study (Soodak and Erwin, 2000) investigated the viewpoints of parents of toddlers with diverse impairments in order to better comprehend the elements that determine their child's inclusive school participation. Based on the data themes, a conceptual framework of factors influencing parent engagement was established. According to the findings, a variety of factors impact parent engagement, including the kindergarten's inclusion vision, responsiveness to parents, and desire to transform. Trust, common views about children and education, and open communication enabled parent-professional interactions. Developing strong parent-professional collaborations in inclusive environments looks to be a difficult process that requires dedication and comprehension. Most significantly, the data indicate that meaningful engagement for both children and parents is a crucial and required component of inclusive education (Soodak and Erwin, 2000).

Parents want their children to socialize socially with classmates who are generally developing, so they enroll them in mainstream early childhood services. Despite greater governmental support for inclusive early childhood education, families encounter various obstacles in locating a center willing and able to fulfill their child's requirements. Parents reported that visiting a mainstream center aided their kid's development, particularly in terms of speech and behavior as a direct outcome participating to qualitative educational activities (Blackmore et al., 2016).

Sylva (2014) intended to connect developmental science findings with pre-school educational research. The study focuses on the importance of the pre-enabling school's environment' in building early executive functions that would subsequently underlie educational performance. According to the results, qualitative preschool education not only created the foundation for academic learning, but also for socio-emotional development, but also cultivated self-control and the executive skills required for long-term planning.

Sira et al. (2018) investigated the perspectives, beliefs, and attitudes of parents of normally developing 3- and 4-year-old children in inclusive preschool classrooms. Using a qualitative technique guided by ecological system theory semi-structured interviews with parents, some common themes related to inclusive preschool interactions were discovered. Parents were less confident in their ability to express the restrictions associated with special needs to their young children, according to the study, even if they supported the principle of inclusion in child care facilities. Developing educational activities and engaging with families and professionals can help to increase parental participation and support in inclusive preschool classes (Sira et al., 2018).

According to study (Zhang and Li, 2021), many parents of children with special educational needs struggle to get intervention and health-care services, or are frustrated. Furthermore, despite a wealth of research on inclusive schooling, scholars have paid less attention to the perspectives of low-income parents on early childhood inclusion. Zhang and Li's research is based on the results of a qualitative survey of 30 New Zealand parents' experiences with early childhood inclusive education. The parents in this research were from various religious backgrounds, ethnicities, had at least one kid with a disability and/or chronic condition, and satisfied New Zealand's low-income criterion. Despite the fact that the majority of families appreciated the flexibility and structure of the early development programs their children attended, parents were concerned about the lack of intervention choices available to them. In addition, these low-income families complained about a lack of early intervention and assistance. The findings also highlight the need of effective coping skills, such as maintaining a good attitude and seeking social support, as well as the relevance of faith in family life. Such coping strategies, in the vast majority of cases, resulted in positive changes and improved the overall wellbeing of families and their impaired children.

### Meso level

The meso-level includes characteristics such as a welcoming atmosphere for each child, a holistic curriculum, an environment for all children, suitable staff credentials, cultural responsiveness, and staff collaboration with families.

Teachers participate in in-and-out-of-school official and informal professional development programs to strengthen their readiness, efficacy, competence, and preparedness for inclusive education, according to qualitative data (Ackah-Jnr, 2020; Ackah-Jnr and Fluckiger, 2021). Motivating teachers, redesigning training sessions and their execution, and supporting teachers' informal learning activities are among the study's recommendations, so that both forms of learning may positively affect teachers' knowledge, skills, and capacities. The fundamental point of the study is that instructors must learn for and about practice. Teachers' personal growth and professional development are vital, and this occurs in a number of situations and styles (Ackah-Jnr, 2018). Professional development paths, also known as learning contexts, are interrelated, and teachers are either internally or externally driven to participate in them. Teachers benefit from professional development to expand their knowledge, comprehension, skill set, attitudinal and perceptual perspective, predisposition, and motivation to perform.

Another recent research (Bjørnsrud and Nilsen, 2019) focused on teachers' perspectives on how collaborative reflection and shared follow-up techniques affect inclusive education growth. The study was carried out using a modified letter technique, in which teachers from one Norwegian school discussed and wrote a collaborative text in response to certain open questions. The findings show that instructors who had previously taken part in a national school development program had formed a wide grasp of inclusive education. They view inclusion to be more than just an issue of placement; it also entails a social and intellectual community. One crucial conclusion is that in a sharing culture, joint reflection produces similar frames of reference for actual inclusion efforts. Teachers' personal experiences imply that collaborative conversation and reflection are equally important in attempts to include students with special educational needs.

Successful inclusive education initiatives are linked to teacher professional development (Ackah-Jnr, 2018). According to studies, formal professional development entails a specific curriculum (Sheehy et al., 2004; West and Pirtle, 2014), specific postgraduate studies (Greene et al., 2020), participation in workshops and conferences (Ofoegbu, 2004; Mitchell and Hegde, 2007; Sheridan et al., 2009), on-the-job training or mentoring (Sheridan et al., 2009; Chuenpraphanusorn et al., 2015; Forlin and Sin, 2017) and on-site school support services (Forlin and Sin, 2017).

Coaching, in-class mentoring, classroom observations, result sharing, and learning exposures are all things that should be considered in professional development activities for teachers to create a comprehensive inclusive education (Forlin and Sin, 2017). According to recent research on preservice teachers, while these young professionals originally expressed fear about working with children with major impairments, their anxiety levels dropped over the practice. The preservice teachers stressed the crucial importance of the pedagogical abilities they received from their mentor. Mentor instructors that are well-qualified in special education were recognized as critical to a great experience (Stites et al., 2021). Peers indicated enjoyment in engaging with and sensitivity toward the disabled youngsters. The findings also revealed that three ways were utilized to facilitate interactions: full involvement of children in activities, modeling responsible behavior, and enlisting the assistance of children who did not have impairments (Hanline and Correa-Torres, 2012).

According to previous research, informal professional learning for inclusive education allows teachers to gain occupational motivation for self-directed instruction and autonomy (Soodak et al., 2002) or to participate in reflective practice (Soodak et al., 2002; Postholm, 2012). Another study's findings point to various implications for practice, including coaches' explicit modeling, reflective practice, and information exchange (Taylor et al., 2022).

Functional diversity training and the production of innovative activities are essential to maximize inclusion. In this educational center's children with and without disabilities, using play and emotions as a foundation for creative projects increases learning, awareness, empathy, and inclusion (Benítez-Lugo et al., 2021). Sustainable competence development, as per Kioupi and Voulvoulis (2019), should include not only cognitive factors such as knowledge and understanding of processes, as well as higher order reasoning ability such as reasoning and synthesizing, but also social skills, value systems, and emotions, collectively known as the affective domain. Open-mindedness, intercultural understanding, and empathy are examples of the former, as are meta-cognitive talents like monitoring processes, which have been shown to influence behavior (Peck et al., 2015; Faham et al., 2017). In another recent qualitative research (Agbenyega and Klibthong, 2021), a thematic analysis revealed 2 themes related to the significance and implementation of inclusion, seeking vital assistance, and a strong desire for a transformative practice (Agbenyega and Klibthong, 2021).

Saloviita (2018) used a large sample of 1,764 instructors to study the views of Finnish elementary school teachers toward inclusive education and discovered that roughly 20% of the teachers were highly opposed to it. Earlier research from Finland (Engelbrecht et al., 2013; Saloviita and Schaffus, 2016) found that Finnish teachers were less receptive of inclusion than their colleagues from Western nations (Avramidis and Norwich, 2002). Numerous instructors rejected inclusive education owing to unpleasant educational experiences, an insufficient support from school officials, and a lack of practical abilities (Bornman and Donohue, 2013; Ahmmed et al., 2014).

Youngsters with and without impairments are regularly participating and learning together in early development activities (Agbenyega and Klibthong, 2014). While many early development educators support children with disabilities' educational rights, there are major barriers to accomplishing these goals in terms of teacher professional competence (Agbenyega and Klibthong, 2022). The study's findings might be used to design effective early childhood teacher professional development programs that will prepare them for successful inclusive practices.

Favorable attitudes have been connected to effective inclusive teaching techniques and good learning experiences in earlier study (Aiello et al., 2017; Sun, 2022). Preschool teachers are crucial in ensuring that all children achieve their maximum development potential (Allan, 2008; Fleer, 2011; Ashman and Elkins, 2012; Jones, 2014; Jones and Gillies, 2014). Preschool teachers take on additional tasks when children with disabilities are included in order to cooperate with interdisciplinary teams to offer high - quality care. Early childhood educators in inclusive schools must do a lot of effort, and they must be supported by parents and school officials.

Children who learn in open, loving, interactive, and supporting contexts outperform their classmates who learn in exclusive, punishing, and stressful situations (Allan, 2008; Allen and Cowdery, 2012; Jones, 2014). Early childhood is crucial for children to develop the healthy social and emotional abilities required for academic and societal success (Haug, 2017).

Zhang (2011) stated that inclusive approaches, personalized educational programs, individualized family service plans, transition arrangements, professional development, and family involvement were all important (2011). Gago-Galvagno et al. (2022) investigated the impact of early childhood education centers on communication, control, and social-emotional development. Infant education has been proven to influence development in disadvantaged situations, which are frequently associated with poor cognitive outcomes. These findings highlight the importance of tackling infant education and social background in order to promote equitable opportunities starting in infancy.

### Macro level

Mainstream availability for all children, national curriculum regulations, teacher preparation for inclusive early childhood education, excellent funding and governance, evaluation and monitoring, and research on inclusive early childhood education comprise the macro level.

Human growth, according to Bronfenbrenner, is impacted by the dynamic interactions between different layers of a person's environment (2005). Although the suggested model focuses on the impact of the educational environment, it is also crucial to include interpersonal connections inside the kindergarten, the functions and structures of the educational institution itself, a child's family setting, and the impact of society in general. Moos (1973) extended on Bronfenbrenner's social ecological model, claiming that the environmental and human components of an educational facility's social ecology had an equal impact on academic and health results. In a kindergarten setting, both of these environmental and human factors models provide good foundations for intervention development (Waters et al., 2009).

According to the Matthew effect in preschool attendance, children who might gain the most from inclusion in high-quality childcare are now the ones who are most likely to be left out. This puts at risk not just its ability to lessen childhood inequality but also the possibility that it will have Matthew repercussions throughout adulthood. Transition from one institution to another, such as from early childhood education to primary school education, tend to duplicate or strengthen inequities since the Matthew effects frequently start before birth. In terms of finances, living circumstances, and parental care, children born into lower income households already have a deficit. They have parents who are less able to help their kids get ready for school than their peers with greater incomes and better levels of education, so they grow up in a less favorable environment for learning (Augustine et al., 2009). This trend of disadvantage building up is accentuated if these kids have less or no access to high-quality preschool education than advantaged kids have. The advantages of high-quality childcare in terms of education preparation and parental employment help better-off children strengthen their competitive edge, but the children who expect to benefit the most are left aside. And when low-income families do succeed to get childcare, they frequently end up using a substandard provider.

In this context, the key conclusion of our scoping research emphasizes the significance of a flexible ECEC curriculum. A curriculum is a necessary tool for fostering mutual understanding and trust among children, teachers, and parents. Its major purpose is to promote learning and development in order to direct ECEC and/or compulsory education activities at the national and local levels. A well-defined curriculum that describes the aims, goals, and processes for early childhood education and care may help practitioners greatly increase their duties in establishing successful learning environments (Oberhumer, 2005; Rayna and Laevers, 2011). A flexible curriculum, on the other hand, may be advantageous to educators since it provides for greater flexibility in planning for children's learning (MacNaughton, 2003). Educational institutes must use a framework approach when developing and improving institutional curriculum based on national curriculum standards.

Teacher qualifications and quality, staff/child ratio, disparity in teacher salaries, high teacher turnover, job demand and supply, and insufficient teacher diversity were all raised in a study on the public-private divide in early childhood teacher education policy in Massachusetts published by Abbate-Vaughn et al. (2011). According to studies, appropriate resources must be made available in order for any policy or program to be implemented efficiently. The parent community and relevant agencies must work together to ensure that children from newborn to 4 years have access to the services they require. Ramps for children in wheelchairs and crutches, teacher aids to assist instructors in dealing with students with disabilities in their classrooms, and ensuring that the teacher-learner ratio is manageable so that all children receive adequate instruction (Bipath et al., 2021).

A recent qualitative thematic analysis revealed 25 subthemes defining the perceived aspects of inclusive early childhood education provision (Bartolo et al., 2021). They were arranged using the structure-process-outcome and ecological systems models. The customized ecosystem model for inclusive early childhood education that resulted contains the following dimensions: inclusive education outcomes, processes, structural elements within the preschool microenvironment, and wider inclusive structural factors at the community and national levels.

Our investigation discovered a variety of structural elements acting at the macro-system level, which agrees with Bartolo's results Bartolo et al., 2016. CARE study findings (Melhuish et al., 2015; Moser et al., 2017; Ulferts et al., 2019) point to similar results. Unitary—-integrated—-systems, early entitlement, appropriate public funding, focused treatment to achieve equitable results, and high quality (as demonstrated by teacher salaries and levels of training) are macro-level characteristics that promote increasing access and adoption of ECEC. It is emphasized that decentralization, bottom-up planning, collaboration between public and commercial providers, and socially engaged organizations improve ECEC outreach, quality, and effectiveness.

The rationale of social investment holds that social policy in modern social democracies should place at least as much emphasis on creating opportunities for the labor markets of today and tomorrow as it does on providing a buffer for protection against social hazards (Van Lancker, 2021). Children and childhood are essential components of any prolific investment strategy in this regard, not only because the public assistance state's ability to continue operating depends on how many and productive future taxpaying individuals there are, but also because childhood disparities are a serious threat to the development of human capital and the main driver of unequal opportunities in the employment market and adulthood. Therefore, effective investment plans need taking action as soon as feasible to counteract the accumulation of advantage.

## Conclusions

**Figure 3:**
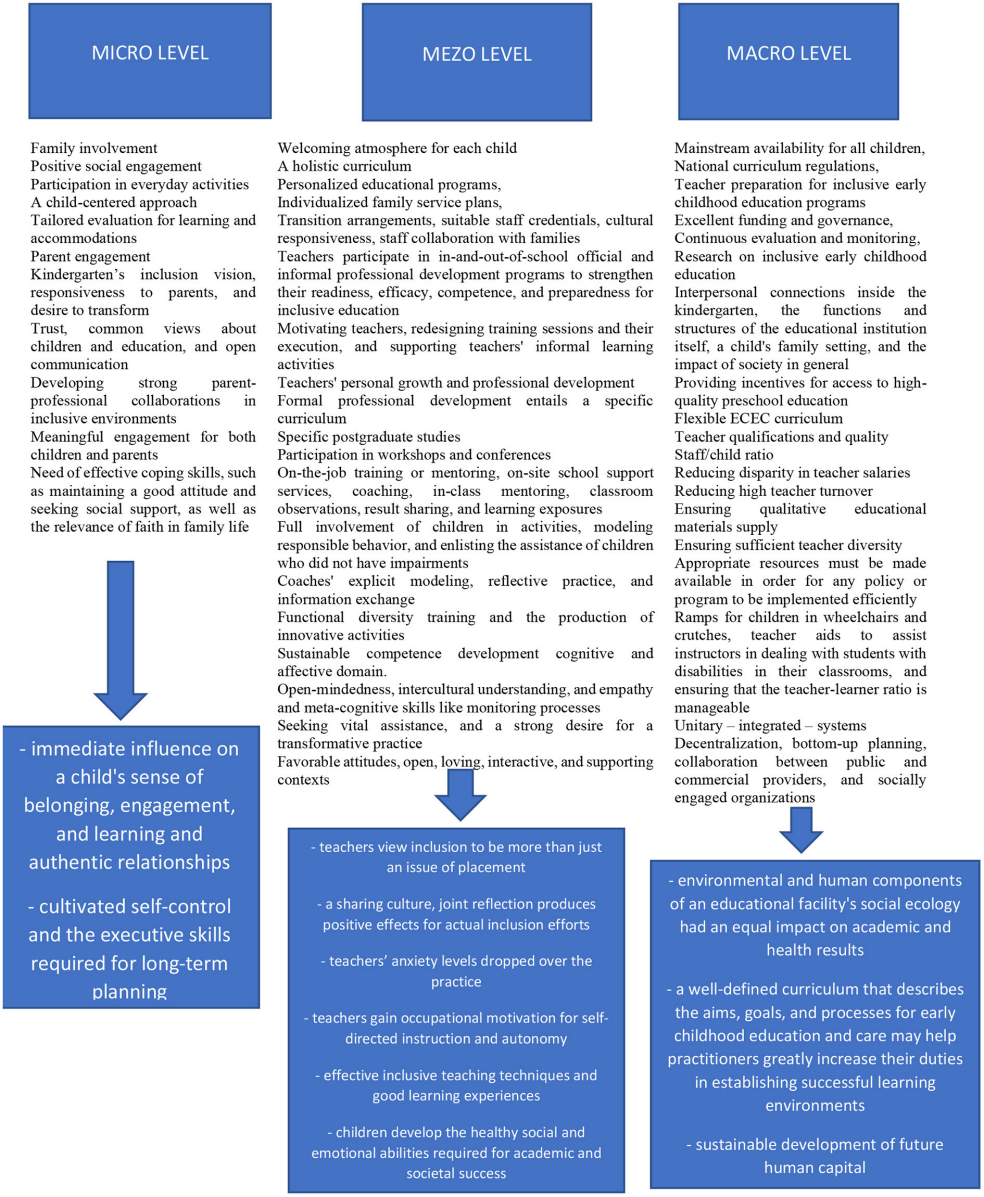
The conceptual framework for implementing inclusive and qualitative ECEC Education.

To summarize the findings of the present scoping review, we are further presenting an overarching figure, as a conceptual framework, which better outlines the diverse influences occurring at the studied levels (i.e., micro, meso and macro). The proposed conceptual framework highlights the forces that contributed to the promotion of a qualitative inclusive early childhood education (Figure 3).

According to studies, inclusive education is critical for children's early development and schooling (Zabeli and Gjelaj, 2020). Inclusion in many facets of a child's growth, including academic, emotional, social, and even brain development, benefits children with special needs. Even yet, it poses significant challenges in terms of pedagogy, particularly when interacting with severe handicaps. Preschool instructors' need for continual professional growth, good training programs, flexible education programs, and the execution of customized educational plans, and a comfortable physical and social environment are all factors that inclusive education must meet; capital abilities and strong institutional support; family and societal support. The findings of the study also revealed a slew of obstacles to achieving inclusive education, many of which are connected to providing the circumstances described above.

The present scoping study points to six important components that are required for implementing inclusive qualitative education in a community undergoing social, economic, and cultural transformations. This model defines and provides a way for inclusive, and qualitative early childhood education, beginning with respect for children's rights and equality for all children and their families, giving them equal access to education in their community's nearby preschool structure, and ending with qualitative inclusive education methodologies promoted by SDG4.2.

**Figure 4:**
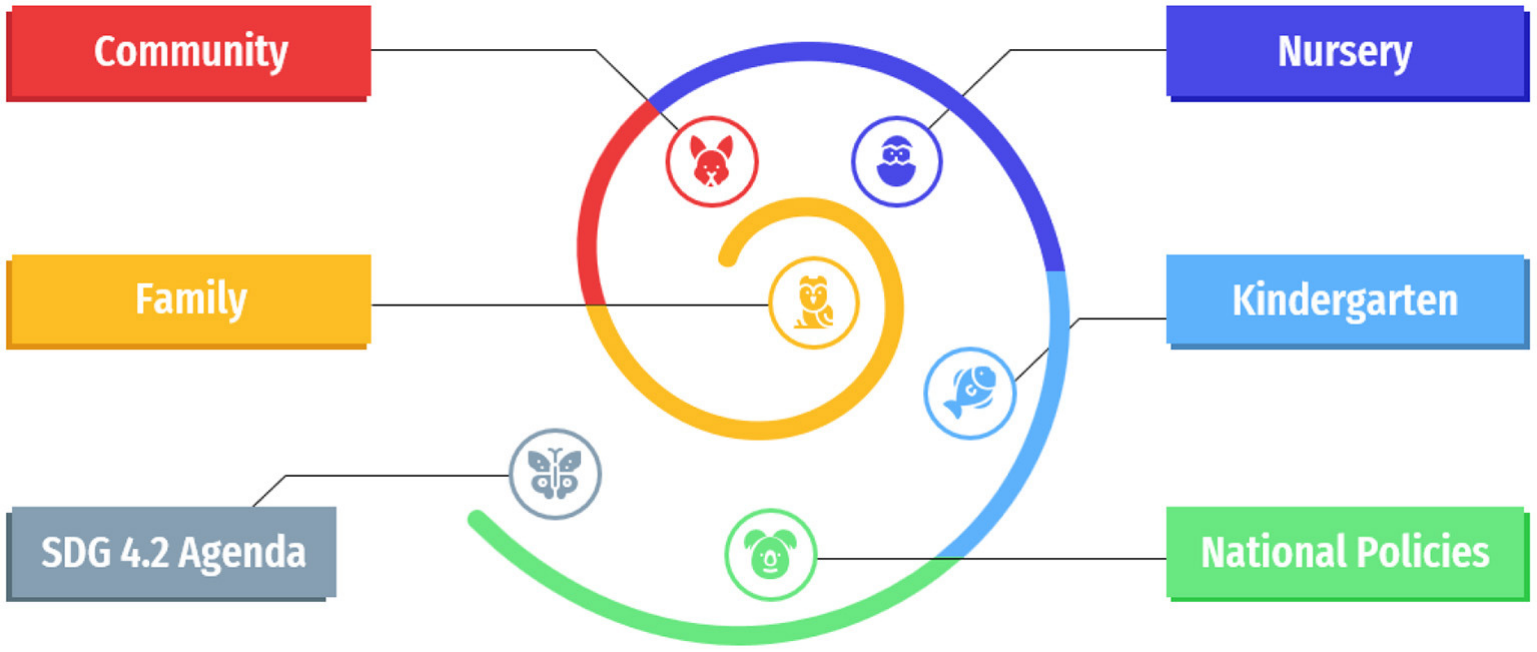
Model of Six layered components for implementing inclusive and qualitative ECEC Education.

In Figure 4, in the spiral infographic diagram we have synthesized the model of six layered components for implementing inclusive and qualitative ECEC strategy starting with the micro level: child, family and community, mezo level: nursery, and kindergarten and macro level: national policies and SDG 4.2 Agenda for 2030. One can notice that the mezzo level connects the micro and macro levels, being the most solicited level of implementing inclusive and qualitative ECEC strategies. Thus, starting with putting a real emphasis on children rights, creating a qualitative and inclusive culture with a holistic understanding of child development, then investing in teacher preparation and instilling a strong belief and positive attitudes toward equity in early childhood services, developing inclusive educational policies with an authentic community support offered by all stakeholders, then adapting curriculum and assessment methods to all early childhood educational contexts and lastly piloting and up-scaling good practices, and investing in infrastructure, facilities and innovative educational services, SDG4.2 targets could transparently and efficiently be attained by 2030, with all the setbacks arisen from the pandemic context.

The data provide light on a vast topic range, including human rights and values, policy actions, and ideologies. Meso-level principles emphasized the relevance of schooling in a child's holistic development and skill acquisition. The micro-level themes emphasized the importance of fostering equitable and inclusive environments for children, as well as instructional approaches that encourage positive attitudes toward diversity and instructors' levels of experience in dealing with diversity. We also discovered the significance of creating chances that promote socialization, connection development, and a sense of belonging.

As a concept and an approach, inclusive and qualitative education necessitates the preparedness of all relevant educational components to participate. In a short amount of time, it is hard to build an inclusive culture and a holistic understanding. This implies that collaboration is essential as part of a process based on previous experiences, research, and practices from around the world. The process of implementing suitable programs, tactics, and assessments, as well as supporting and monitoring teachers, in order to guarantee that all students receive an excellent education (Piškur et al., 2021). Providing inclusive education in the early years requires setting the foundation for subsequent levels of schooling. The active engagement of a young kid should be directed by developmentally and individually suitable curricula. Access to and participation in age-appropriate general curricula becomes critical in identifying and providing specialized support services.

## Limitations

Scoping reviews, which are used to categorize or organize the body of literature in a field, such as qualitative and inclusive ECEC strategies for achieving SDG4.2 target, based on its nature, characteristics, and volume, are extremely useful for synthesizing research data. Grant and Booth (2009) define scoping reviews as a first evaluation of the prospective size and extent of the body of research literature that is already accessible aims to define the type and scope of research evidence, which often includes active research. When an amount of literature has not yet had a thorough evaluation or is too broad, complicated, or diverse to be subjected to a more focused systematic review, scoping reviews are the best option.

A scoping review makes it simpler to compile an overview of the state of the field's existing knowledge, identify knowledge gaps, and direct future research on the topic. The method is suitable for this investigation due to its openness and rigor. Flexibility, a broad scope, and the incorporation of gray literature were difficulties that were also strengths of scoping studies since they made it difficult to draw limits for the study's scope.

Scoping research were frequently time-consuming and took longer than initially anticipated because of the vast reach and unclear limits, which left an excessive quantity of data. This might increase the chance that a scoping study will rapidly become outdated. Since the goal of scoping studies is to map the evidence generated in a particular subject rather than seek for the best evidence available to address a topic connected to policy and practice, the absence of methodological quality evaluation is to be seen as a distinctive feature of these studies (Daudt et al., 2013; O'Brien et al., 2016). In general, the growing variety of knowledge syntheses may make it unclear whether or not scoping research should be conducted. When deciding between a scoping study and another sort of knowledge synthesis, it is crucial to take the research question, the field's context, and the type and quality of evidence available into account (Peters et al., 2015). Scoping reviews frequently collect data using a variety of research designs and methodologies without officially evaluating the quality of the evidence, like in the case of current scoping review on qualitative and inclusive E|CEC strategies for attaining SDG4.2.

By design, a large number of research were included in the present review process. As a result, screening the numerous papers and other sources for possible inclusion in the scoping review required a large research team. Because the current scoping reviews provides a descriptive picture of the literature that is already accessible, this resulted in broad, less specific searches, like the methodological steps presented in the methodology section. The present scoping review offers an overview of the body of research rather than a summarized outcome. Like other scoping reviews, this review is susceptible to selection bias due to the vast amount of investigated literature.

## Implications

### Implications for practice

Inclusive programming does not imply that the educational programs will necessarily be of good quality (Taylor et al., 2022).

Efficiency and wellbeing are synonymous with equity (Milovantseva et al., 2018). Equitable education investment benefits everyone in society, not just the most marginalized (Tatto, 2021). Investing in education will help communities achieve all of the Sustainable Development Goals related to education.

Education is essential for escaping persistent poverty (Herrmann and Rundshagen, 2020; Nakidien et al., 2021). Poverty is only temporary for some individuals. However, the most vulnerable people stay impoverished for lengthy periods of time, if not their whole lives, passing on their poverty to their offspring. Education prevents poverty from being passed down through generations. Designing and implementing transformational public policies in response to the variety and needs of learners, as well as addressing the many types of discrimination and conditions, including crises, that obstruct the realization of the right to education, is crucial.

In keeping with the wider 2030 Agenda for Sustainable Development, cross-sector policies and programs should be adopted or extended to remove the social, cultural, and economic barriers that hinder millions of children, youth, and adults from receiving excellent education (Boeren, 2019; Friedman et al., 2020). Removal of financial obstacles through cash transfer programs, the availability of school meal options, and the provision of healthcare services are instances of evidence-based policies and measures to combat exclusion, along with teaching and learning resources, as well as transportation services; second-chance programs; inclusive school infrastructures; inclusive teacher training; and language policies.

ICTs have the potential to be a powerful tool for social inclusion (Lorente et al., 2020). All children and youngsters, regardless of their unique characteristics or circumstances, have the potential to achieve excellence and contribute to the society and this must be stressed. Families and communities must be more fully involved in holistic education in order to achieve this (Hanline and Correa-Torres, 2012; Roux et al., 2012; Sylva, 2014; Meeks and Jain, 2015; McDevitt, 2021).

Early Childhood Education and Care is a high-return investment in individual growth and wellbeing, as well as the peaceful development and stability of society (Nelson and Carver, 1998; Enlow et al., 2012; Ilie and Rose, 2016; Chaleta et al., 2021; Hueske et al., 2021; Nakidien et al., 2021).

Education funding should be a top priority for those who are most in need. Vulnerable adolescents in crisis zones frequently have the highest educational needs, and funds should be allocated toward them (Deng, 2003; Kreso, 2008; Siriwardhana et al., 2013; Pascapurnama et al., 2018). Financing should be tailored to their specific requirements and based on previous experience.

Methods for inclusive decision-making are critical to ensure that no one is left behind (Beeharry, 2021). Over the next 15 years, decision-making procedures are projected to become more democratic, with people's perspectives and goals represented in the creation and implementation of education policy at all levels (Addey, 2021; Shabalala and Ngcwangu, 2021). Strong, multi - faceted collaborations that bring together all key stakeholders, such as governments, non-governmental organizations, teaching staff and education professionals, parents and families, the private sector, charitable organization, the research community, and youth, students, and their organizations, can aid in planning, implementation, and monitoring (Sunarti and Zukdi, 2019; Lakkala et al., 2020).

To summarize, we support Brissett and Mitter (2017) caution against the excitement surrounding their adoption, as uncritical acceptance of its form and substance may just perpetuate the global social status quo of unfairness. Ultimately, this would need a shift away from easy-to-measure metrics like teacher certifications and toward more meaningful and valid indices like teacher qualities, a disregarded topic in the SDG4 objectives (Tatto, 2021). SDG 4 requires a shift in prevailing educational rhetoric; the goals of education must be widened so that quality education is no longer just connected with uniformity, efficiency, and employment, but is instead being regarded as a fundamental human right and a catalyst for social change. Education must be recognized and used as a tool to discover and rectify societal inequities, with social and environmental justice at the forefront (Coombs, 1994; Gheorghiu et al., 2021).

### Implications for academicians

Suggestion for future research imply a multilevel analytics perspective over the effectiveness of ECEC practices, in order to counterbalance the Matthew effect, a social effect that is closely related to fairness concerns (Van Lancker, 2021).

Investments in childcare will have the reverse of the desired effect if low-income families cannot access it. Better-off people are more likely to use and gain from the assistance that is offered, which increases their advantage and widens the gap. In ECEC, this phenomenon—in which the wealthy continue to become richer and the poor remain poor—is known as the Matthew Effect. Better-off families likely to benefit from current government initiatives meant to increase daycare accessibility. Supporting parents pay for childcare so they can work is one goal of the childcare system. However, daycare usage is related to how much parents work and, therefore, how much profit they earned. The more childcare they use, the more help they are likely to get. Children from wealthy households may benefit more from this, whereas children from low-income families may lose out. To ensure there are adequate childcare spaces, that these spaces are inexpensive, accessible to low-income families, and of a good quality, deliberate investment is needed in childcare policy. This indicates that the kids who would gain the most from being included in high-quality childcare are the ones who are now most likely to be left out. This might potentially drive compounding inequities throughout the course of a person's life, putting at risk the ability of childcare services to lessen inequalities in early life. For instance, reducing childhood poverty may prevent or delay the development of cumulative disadvantage.

According to studies, welfare states that are more generous and egalitarian are better at preventing the intergenerational transfer of poverty and enhancing the educational and employment prospects of children from low-income households (Corak, 2013). Even yet, it is still unclear exactly how the Matthew effect is affected by the combination of agency and policy structure throughout the course of a person's life. Fundamentally, rather than the actual mechanisms behind these processes, most study focuses on the results of the Matthew effect, or the aggregation of several Matthew effects occurring in various organizations (DiPrete and Eirich, 2006). In any event, the research implies that the Matthew effect is substantially influenced by policy rather than being an unavoidable feature of reality. The design and execution of policies, however, will determine how they impact the Matthew effect.

## Data availability statement

The original contributions presented in the study are included in the article/supplementary material, further inquiries can be directed to the corresponding authors.

## Author contributions

All authors listed have made a substantial, direct, and intellectual contribution to the work and approved it for publication.

## Funding

This study was funded by Academia Oamenilor de Ştiinţă din România, with support from Romanian Ministry of Education during the implementation of the National project *Qualitative and Inclusive Early Childhood Education and Care* and collaboration from Romanian School Inspectorates and the preschool instructors who participated in our initiative from the beginning.

## Conflict of interest

The authors declare that the research was conducted in the absence of any commercial or financial relationships that could be construed as a potential conflict of interest.

## Publisher's note

All claims expressed in this article are solely those of the authors and do not necessarily represent those of their affiliated organizations, or those of the publisher, the editors and the reviewers. Any product that may be evaluated in this article, or claim that may be made by its manufacturer, is not guaranteed or endorsed by the publisher.
